# New Perspectives in the Renin-Angiotensin-Aldosterone System (RAAS) I: Endogenous Angiotensin Converting Enzyme (ACE) Inhibition

**DOI:** 10.1371/journal.pone.0087843

**Published:** 2014-04-01

**Authors:** Miklós Fagyas, Katalin Úri, Ivetta M. Siket, Andrea Daragó, Judit Boczán, Emese Bányai, István Édes, Zoltán Papp, Attila Tóth

**Affiliations:** 1 Division of Clinical Physiology, Institute of Cardiology, University of Debrecen, Debrecen, Hungary; 2 Department of Neurology, University of Debrecen, Debrecen, Hungary; 3 Institute of Internal Medicine, Division of Nephrology, University of Debrecen, Debrecen, Hungary; School of Pharmacy, Texas Tech University HSC, United States of America

## Abstract

Angiotensin-converting enzyme (ACE) inhibitors represent the fifth most often prescribed drugs. ACE inhibitors decrease 5-year mortality by approximately one-fifth in cardiovascular patients. Surprisingly, there are reports dating back to 1979 suggesting the existence of endogenous ACE inhibitors, which endogenous inhibitory effects are much less characterized than that for the clinically administered ACE inhibitors. Here we aimed to investigate this endogenous ACE inhibition in human sera. It was hypothesized that ACE activity is masked by an endogenous inhibitor, which dissociates from the ACE when its concentration decreases upon dilution. ACE activity was measured by FAPGG hydrolysis first. The specific (dilution corrected) enzyme activities significantly increased by dilution of human serum samples (23.2±0.7 U/L at 4-fold dilution, 51.4±0.3 U/L at 32-fold dilution, *n* = 3, *p* = 0.001), suggesting the presence of an endogenous inhibitor. In accordance, specific enzyme activities did not changed by dilution when purified renal ACE was used, where no endogenous inhibitor was present (655±145 U/L, 605±42 U/L, *n* = 3, *p* = 0.715, respectively). FAPGG conversion strongly correlated with angiotensin I conversion suggesting that this feature is not related to the artificial substrate. Serum samples were ultra-filtered to separate ACE (MW: 180 kDa) and the hypothesized inhibitor. Filtering through 50 kDa filters was without effect, while filtering through 100 kDa filters eliminated the inhibiting factor (ACE activity after <100 kDa filtering: 56.4±2.4 U/L, *n* = 4, control: 26.4±0.7 U/L, *n* = 4, *p<0.001*). Lineweaver-Burk plot indicated non-competitive inhibition of ACE by this endogenous factor. The endogenous inhibitor had higher potency on the C-terminal active site than N-terminal active site of ACE. Finally, this endogenous ACE inhibition was also present in mouse, donkey, goat, bovine sera besides men (increasing of specific ACE activity from 4-fold to 32-fold dilution: 2.8-fold, 1.7-fold, 1.5-fold, 1.8-fold, 2.6-fold, respectively). We report here the existence of an evolutionary conserved mechanism suppressing circulating ACE activity, *in vivo*, similarly to ACE inhibitory drugs.

## Introduction

Angiotensin-converting enzyme (ACE) is a member of the renin-angiotensin-aldosterone system (RAAS), which is an important regulator of blood pressure and salt-water homeostasis [1]. It is a zinc-metalloendodipeptidase with two catalytically active sites (N- and C-terminal catalytic domains). ACE catalyzes the conversion of angiotensin I to angiotensin II, and the metabolism of other peptides such as bradykinin [2].

ACE inhibitor drugs are the fifth most frequently prescribed drugs [3], there were 162.8 million ACE inhibitor prescriptions in 2009 in the United States of America alone [3]. Their effectiveness has been demonstrated by several large clinical trials: ACE inhibitors reduce the risk of cardiovascular death, nonfatal myocardial infarction or cardiac arrest in stable coronary heart disease [4], improve the prognosis [5] and reduce the 5-week mortality after myocardial infarction [6], reduce heart failure mortality [7], inhibit left ventricular remodeling [8], delay the manifestation of hypertension [9], and reduce the left ventricular mass index in left ventricular hypertrophy [10], the incidence of microalbuminuria and the risk of diabetic nephropathy in type 2 diabetes [11] and the likelihood of newly diagnosed diabetes mellitus [12]. The latest therapeutic guidelines have incorporated all these features [13]–[18]•••, and ACE inhibitors are considered to be an important component of the polypill proposed as a means of reducing cardiovascular disease by 80% [19].

Interestingly, there are several reports suggesting the existence of endogenous ACE inhibitors dating back to 1979. It has been reported that human serum albumin [20] and small (<10 kDa) molecules [21] can inhibit ACE. Several more inhibitors has been suggested afterward. These included substrate analogues [22], [23] and various other proteins [24], [25]. Consequently efforts were made to isolate and identify inhibitory molecules associating with ACE in the rat lung [26] or in human sera [27], but these efforts were overshadowed by the profound clinical success of ACE inhibitory medication.

The goal of this present work was to study endogenous ACE inhibition in human sera. It was found that the circulating ACE is substantially inhibited by a non-competitive endogenous inhibitor. Our data suggest that circulating ACE activity is regulated *in vivo*, an effect which is similar to ACE inhibitory medication in cardiovascular disease.

## Methods

### Ethical approval

All of the studies were approved by the Regional and Institutional Ethics Committee, Medical and Health Science Center, University of Debrecen, (UDMHSC REC/IEC number: 2894-2008) and by the Medical Research Council of Hungary. All of the patients involved gave their written informed consent.

### Patient's blood sample collection, serum isolation

Blood samples were collected from volunteers by using a standard aseptic technique. Native blood was incubated for 60 minutes at room temperature; serum fractions (separated by centrifugation at 1,500 *g* for 15 min) were stored at −20°C until further experiments.

### ACE activity measurement using spectrophotometric assay

ACE activity was measured as described by Beneteau et al. [28]. In brief, ACE activity was determined with an artificial substrate (FAPGG, (*N*-[3-(2-furyl)acryloyl]-L-phenylalanylglycylglycine; Sigma-Aldrich) in a reaction mixture containing 25 mM HEPES (*N*-2-hydroxyethylpiperazine-*N*-2-ethanesulfonic acid), 0.5 mM FAPGG, 300 mM NaCl, and the desired dilution of serum, at pH 8.2. Measurements were performed in 96-well plates (Greiner-Bio One) at 37°C. Changes in optical density (340 nm) were measured at 5-min intervals for at least 90 min with a plate reader (NovoStar plate reader; BMG Labtech). Optical density values were plotted as a function of reaction time and fitted by linear regression (GraphPad Prism 5.0). The fit and the data were accepted when *r^2^* was >0.90. ACE activity was calculated via the equation:
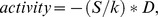
where *S* is the rate of observed decrease in optical density (1/min), *k* is the change in optical density upon the complete cleavage of 1 µmol of FAPGG, and *D* is the dilution of the serum. ACE activity is given in units where 1 U is equivalent to the cleavage of 1 µmol of FAPGG in 1 min.

### Measurement of domain specific ACE activity

Domain specific ACE activity was measured as described by Carmona et al. [29]. In brief, quenched fluorescent peptide substrates were used. Abz-SDK(Dnp)P-OH (Sigma-Aldrich) is highly specific for N domain active site, Abz-LFK(Dnp)-OH (Sigma-Aldrich) for C domain active site and Abz-FRK(Dnp)P-OH (Sigma-Aldrich) can be cleaved by both active sites. The reaction mixtures contained 100 mM tris(hydroxymethyl)aminomethane hydrochloride (TRIS HCl, Sigma-Aldrich), 50 mM NaCl, 10 µM ZnCl2 and 40 µM Abz-SDK(Dnp)P-OH or 50 µM Abz-LFK(Dnp)-OH or 10 µM Abz-FRK(Dnp)P-OH fluorescent substrate, and the serum samples, at pH 7.0. Measurements were performed in black, 96-well plates (Greiner-Bio One) at 37°C, λex was 340 nm, λem was 405 nm. Changes in fluorescence intensities were measured at 4-min intervals in case of domain specific substrates for at least 90 min, and at 1.5-min intervals in case of Abz-FRK(Dnp)P-OH substrate for at least 30 min with a plate reader (NovoStar plate reader; BMG Labtech). Fluorescence intensity values were plotted as a function of reaction time and fitted by a linear regression (GraphPad Prism 5.0). The fit and the data were accepted when *r^2^* was >0.95. ACE activity was calculated via the equation:
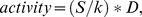
where *S* is the rate of observed increase in fluorescence intensity (1/min), *k* is the change in fluorescence intensity upon the complete cleavage of 1 µmol of fluorescent substrate, and *D* is the dilution of the sample. ACE activity is given in units where 1 U is equivalent to the cleavage of 1 µmol of fluorescent substrate in 1 min.

### Direct measurement of ACE-catalyzed angiotensin I conversion

Serum samples containing 0.5 µM angiotensin I (GenScript) and 300 mM NaCl in 25 mM HEPES buffer, pH 8.20 were incubated at 37°C. 5 mM EDTA was added to stop the reaction. Angiotensin peptides were measured after filtering through a filter with a 10 kDa pore size (Vivaspin, Sartorius Stedim Biotech). HPLC analysis was performed with a HPLC technique on a reverse-phase C18 column (Hypersil Gold, Thermo Scientific). Eluent A was 0.01% aqueous trifluoroacetic acid (TFA, Sigma-Aldrich), while eluent B was 0.01% TFA in acetonitrile (Sigma-Aldrich). Angiotensin peptides were separated by using an elution profile with a gradient from 22% acetonitrile to 55% acetonitrile. They were detected by a diode array detector at 230 nm and the area under the curve of each angiotensin peptide peek was compared with calibration curves recorded when the purified peptide was tested. The amounts of angiotensin peptides were plotted against the reaction time and fitted by linear regression. The kinetics of angiotensin I conversion was multiplied by the dilution of the sera and given in µmol angiotensin I cleavage in 1 L of serum in 1 min.

### Fractionation of human sera

Serum samples from a healthy volunteer were ultrafiltered through ultrafiltration devices with a pore size of 50 or 100 kDa (Vivaspin 500, Sartorius Stedim Biotech) at 4°C for 6 min at 15,000 *g*. One volume of initial serum sample was diluted to 250-fold in 25 mM HEPES, pH 8.2 (yielding 250 volume of diluted serum sample). Then diluted serum samples were ultrafiltered until the retained volumes reached the initial volumes of the serum samples (1 volume). ACE concentrations of an initial serum sample and the retained sample were compared to evaluate the potential loss (or enrichment) of ACE using a human ACE ELISA kit (R&D Systems). No difference was found between the ACE concentration in the initial sample and in the retained fraction after filtration (pore size: 100 kDa, ACE concentration: 119.5±12.0 ng/mL, n = 3 and 111.8±11.0 ng/mL, n = 3, p = 0.459, respectively), suggesting maintained concentrations of the proteins above the pore size limit of the membranes.

### Statistical analysis

Statistical analysis was performed with Graphpad Prism 5.0 software (GraphPad Software) by unpaired t-tests. Differences were considered to be significant when *p*<0.05.

## Results

**Figure 1:**
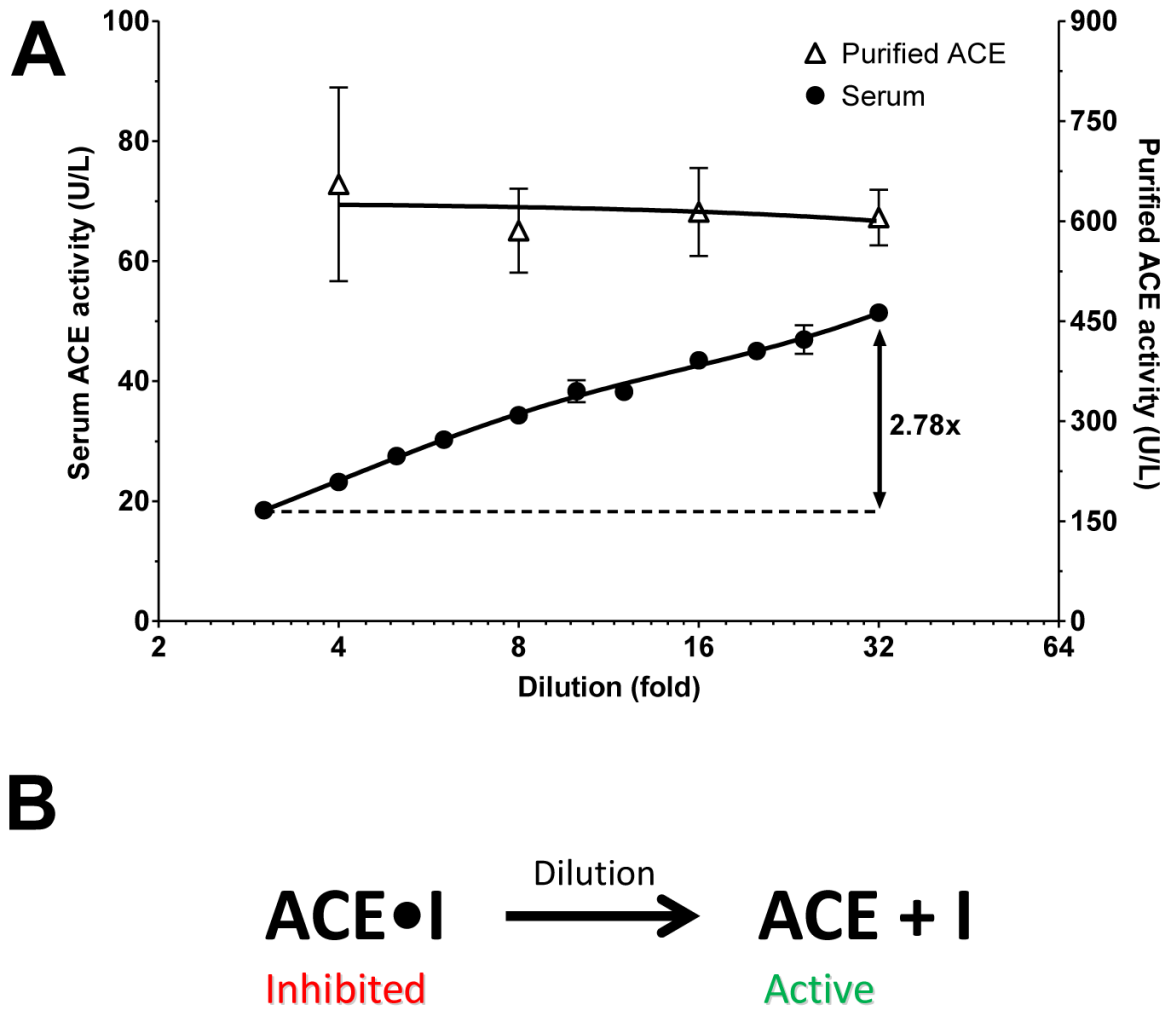
Endogenous inhibitor masks ACE activity in human sera. Effects of dilution on the ACE activity were tested on purified renal ACE (Purified ACE) and human serum (**A**). Plots were fitted by means of linear regressions in case of purified ACE while nonlinear regression was used in case of serum ACE (indicated by the solid lines), while symbols denote the means ± SEM of at least 3 independent determinations. The increase of specific serum ACE activity (2.78-fold) upon dilution is also indicated. The hypothesis proposed to explain these findings is that an endogenous inhibitor is present in the sera, which inhibits ACE at low dilution and then dissociates at higher dilution, unmasking ACE activity (**B**).

Serum ACE activity was significantly affected by dilution (Fig. 1A), increasing from 18.5 U/L at a 3-fold dilution to 51.4 U/L at 32-fold dilution. In contrast, no such effects of dilution were found on the purified tissue ACE activity (Fig. 1A). These data suggest that ACE activity is modulated by additional factors besides the ACE itself in the human serum. We hypothesized that an endogenous reversible inhibitor is present in the human sera. According to this hypothesis the endogenous reversible inhibitor dissociates from the ACE at high dilutions, resulting in an apparent increase in specific activity (Fig. 1B).

**Figure 2:**
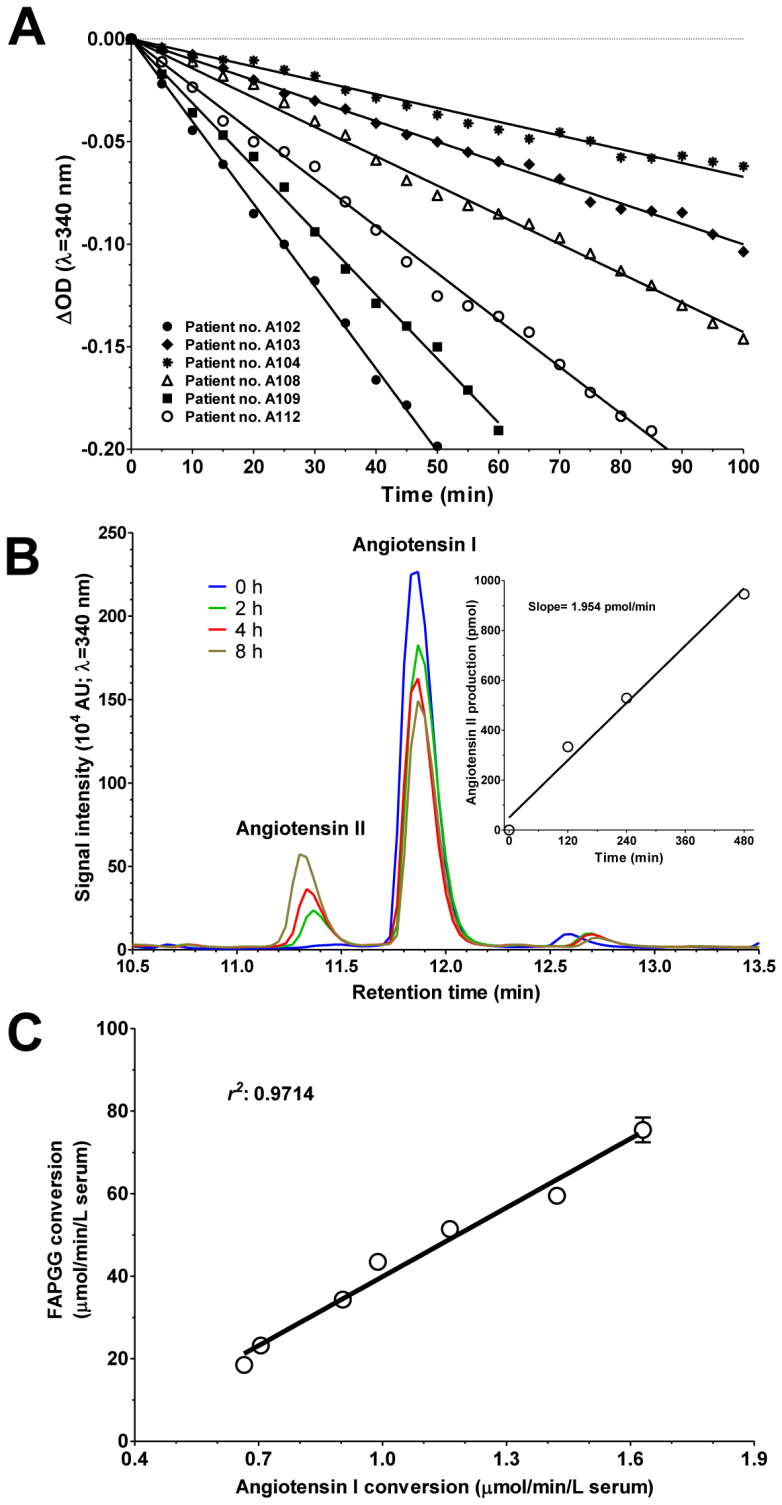
FAPGG hydrolysis proportional to angiotensin I conversion. A representative ACE activity measurement is shown in **A**. Decomposition of FAPGG results in a decrease in optical density at 340 nm. The decreases in optical density were plotted as a function of the reaction time and fitted by linear regression. The kinetics of decomposition was followed for at least 90 min. The slope of the linear regression was taken as a measure of the ACE activity. Determinations were repeated at least 3 times to obtain activity values. The ACE activity was also determined by using its endogenous substrate angiotensin I, which is converted to angiotensin II by ACE (**B**). This reaction was followed by HPLC. Area under the curve was calculated to quantify the amount of the peptides. Calibration plots were constructed based on standard concentrations of the peptides. Angiotensin I conversion was followed by a kinetic assay, where angiotensin I and angiotensin II were determined after 0, 2, 4 and 8 h. The slope of the linear fit gave the ACE activity. A single representative experiment is illustrated (**B**). Parallel experiments were performed to compare FAPGG and angiotensin I-converting activities of the same sample (**C**). FAPGG converting activities were plotted as a function of angiotensin I converting activities. Plots were fitted by means of linear regression (solid line), the symbols denote means ± SEM of 3 independent determinations. The goodness of fit (*r^2^*) is also indicated on figure.

The cleavage of the artificial substrate *N*-[3-(2-furyl)acryloyl]-L-phenylalanylglycylglycine (FAPGG, determined photometrically, Fig. 2A) by human sera was compared with the conversion of the physiological substrate angiotensin I (determined by HPLC, Fig. 2B), *in vitro*, in separate sets of experiments. ACE activity determined by FAPGG cleavage was directly proportional to angiotensin I to angiotensin II conversion (Fig. 2C), albeit FAPGG cleavage was about 30-fold faster and easier to measure.

**Figure 3:**
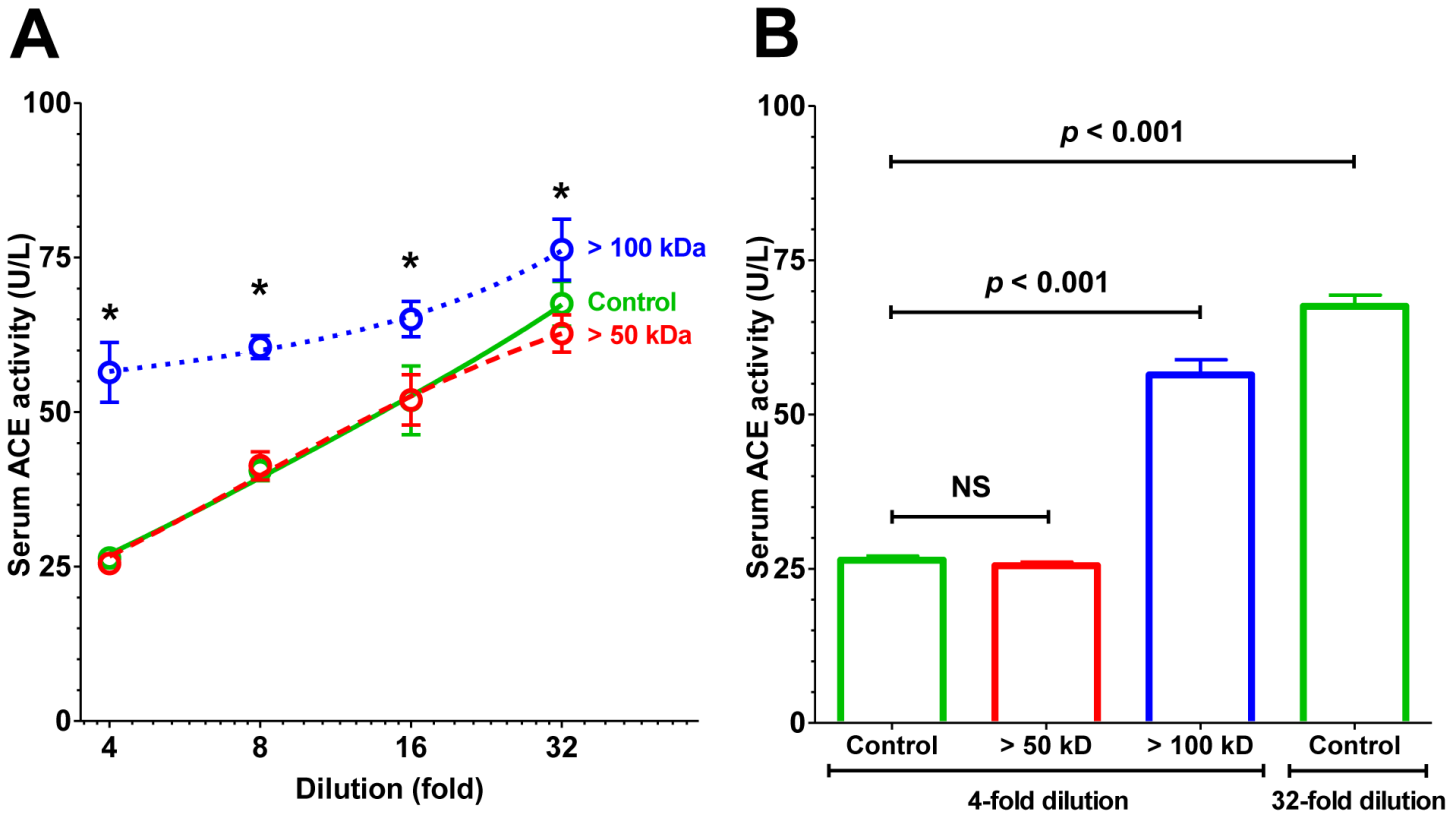
The size of the endogenous ACE inhibitor is in the range of 50–100 kDa. To determine the size of the endogenous ACE inhibitor, serum samples were filtered through filter devices (50 kDa pore size, red; and 100 kDa pore size, blue; **A**). 1 volume of serum was diluted to 250 volume by the buffer and then ultrafiltration was done until 249 volume of the diluted sample has flown through the indicated pore size membranes, yielding 1 volume of retained fraction (being the same as the volume of the initial serum sample). The ACE concentration was the same in the initial serum sample and in the retained fraction, suggesting maintained protein concentrations in the case of the proteins with higher molecular sizes than the pore size of the membranes (50 and 100 kDa). Both the initial sera and the retained fractions were diluted to the same extent to be able to compare ACE activities. Since ACE concentration was the same in these samples at any dilutions, the effects of filtration is the consequence of the loss of the inhibiting factors upon filtration. At least 4-fold dilution of the sera was necessary to measure ACE activity by FAPGG in the initial sera (high level of absorbance at 340 nm by the human serum itself), therefore both the initial samples and the retained fractions were diluted to the same level (4-fold) to compare the inhibited activities. Similarly, both samples were diluted to 32-fold to estimate the level of maximal ACE activity, when the inhibitor was dissociated. Symbols denote means ± SD of 4 independent determinations. Significant differences from the unfiltered serum (green, **A**) are indicated by asterisks. Serum ACE activities are also shown on the bar graph (**B**) at 4-fold dilution before (Control, green) and after filtering through 50 kDa (red) and 100 kDa pore size (blue) devices. Unfiltered serum ACE activity is also shown at 32-fold dilution (Control, green). Bars denote means ± SEM of 4 independent determinations, significant differences are indicated by *p* values.

Efforts were made to prove the presence of the endogenous inhibiting factor. Serum samples were filtered through ultrafilter devices with different pore sizes. No effect was observed when proteins with <50 kDa molecular masses were removed, suggesting that the inhibitor is above 50 kDa (>50 kDa, Fig. 3A). In contrast, depletion of proteins with molecular masses <100 kDa from the human serum resulted in significantly elevated ACE activities, without the apparent gradual increase in ACE activity seen upon dilution in the original serum samples (fraction above 100 kDa, >100 kDa, Fig. 3A). In particular, the ACE activity determined at the lowest (4-fold) dilution was ∼2.2-fold higher in serum depleted of proteins with molecular masses <100 kDa (increase in specific ACE activity from 26.4±0.7 U/L to 56.4±2.4 U/L, fraction >100 kDa, Fig. 3B). Moreover, serum samples depleted from the endogenous inhibiting factor had similar activities at 4-fold dilution than that for unfiltered serum samples after dilution to 32-fold (at which conditions the endogenous inhibitor is dissociated from the ACE, 56.4±2.4 U/L, 67.5±1.8 U/L, respectively, Fig. 3B).

**Figure 4:**
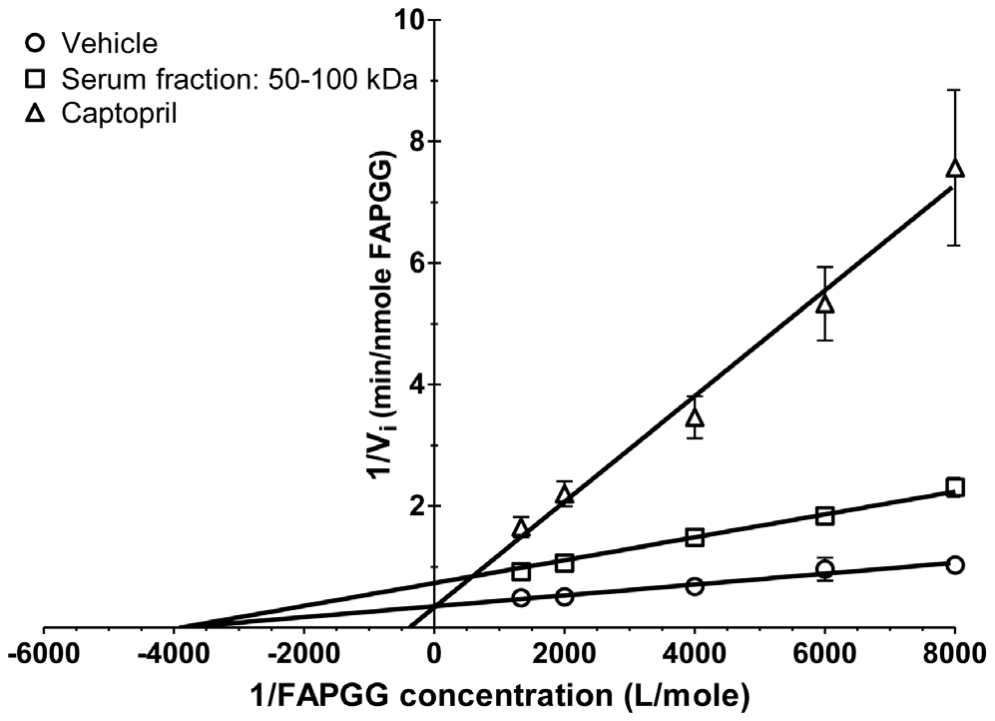
Non-competitive ACE inhibition by the endogenous serum factor. The reaction kinetics of FAPGG hydrolysis (in nmole/min units) was determined at different FAPGG concentrations (750, 500, 250, 167 and 125 µM) to create a Lineweaver-Burk (double reciprocal) plot. The same experiments were performed in the absence (vehicle) and presence of captopril (50 nM) and the 50–100 kDa fraction of the human serum (4.5-fold dilution). Symbols represent means ± SEM of 3 independent determinations. Values were fitted by a linear regression.

The type of inhibition was addressed next. ACE activity was measured at constant inhibitor concentrations (serum fraction, containing the endogenous inhibitor, 4.5-fold diluted compared to the initial concentration of the 50–100 kDa components in the human sera; captopril, an ACE inhibitory drug, 50 nM) using different concentrations of the substrate (FAPGG, Fig. 4). A Lineweaver-Burk plot was designed showing competitive ACE inhibition by captopril (note similar y-axis intercepts in the cases of vehicle and captopril), while the inhibition was found to be non-competitive in the presence of the serum fraction (note similar x-axis intercepts in cases of vehicle and endogenous serum inhibitor).

**Figure 5:**
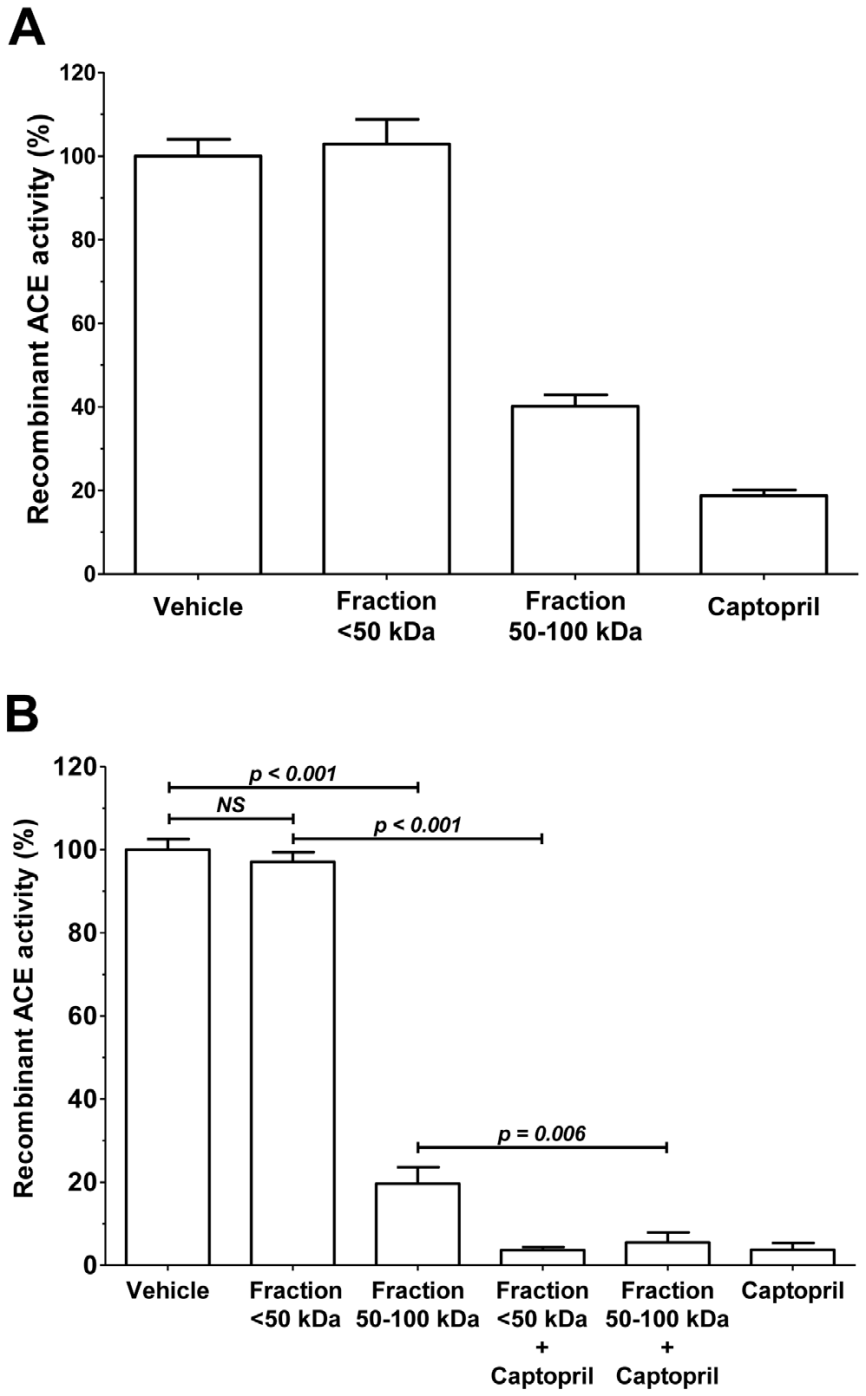
Efficacy of the ACE inhibition by human serum fractions and captopril. Inhibition of recombinant ACE was measured alone (vehicle) or in the presence of four-fold diluted human serum fractions containing components below 50 kDa (<50 kDa), in the range of 50–100 kDa and 1 µM captopril. Experiments were done first using the physiological substrate angiotensin I (**A**). Bars represent means ± SD of 2 independent determinations, values are expressed in the percentage of vehicle. The effects were also determined using the artificial substrate FAPGG (**B**). In this latter case serum fractions were also combined with captopril (<50 kDa+captopril and 50–100 kDa+captopril), in addition to the above mentioned conditions (<50 kDa, 50–100 kDa and 1 µM captopril). Bars represent means ± SD of 3 independent determinations, values are given in the percentage of vehicle, significant differences are indicated by *p* values.

Captopril had higher efficacy than that is for 4-fold diluted endogenous human serum inhibitor (50–100 kDa fraction) on recombinant ACE catalyzing angiotensin I to angiotensin II conversion measured by HPLC, while no inhibitory effect was detected in the case of serum proteins below 50 kDa (<50 kDa, Fig. 5A). The same was noted in FAPGG hydrolysis (Fig. 5B). Moreover, inhibitory effect of captopril was not affected by <50 kDa or 50–100 kDa serum fractions (Fig. 5B). Combination of captopril and the endogenous serum inhibitor (50–100 kDa fraction) resulted in higher ACE inhibition, than that is in the absence of captopril (Fig. 5B).

**Figure 6:**
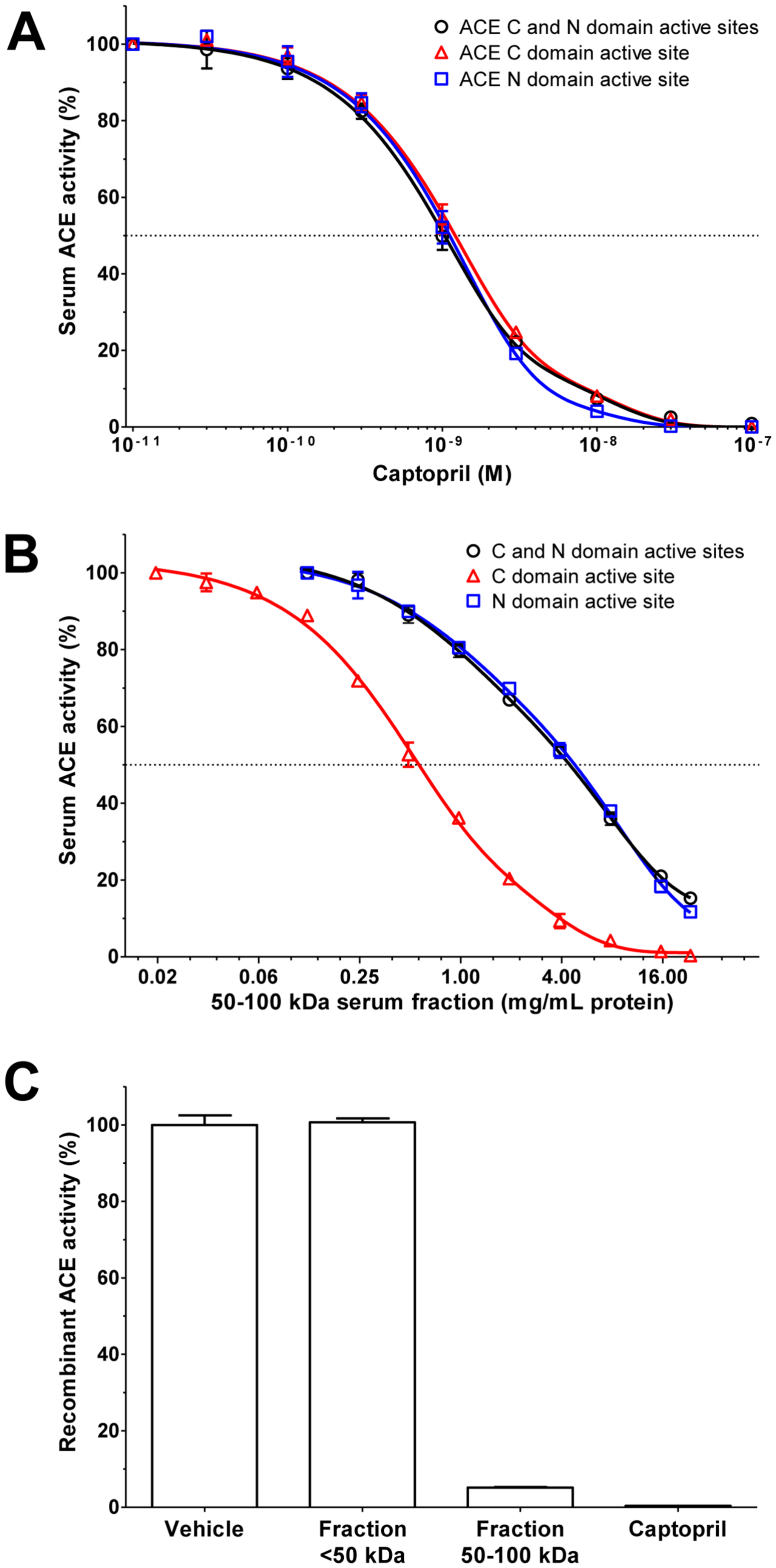
The endogenous ACE inhibitor has higher affinity at the C-terminal active site of ACE. Inhibition of serum ACE was tested by active site specific flourescent substrates: (Abz-SDK(Dnp)P-OH (triangles) for the N-terminal active site, Abz-LFK(Dnp)-OH (squares) for the C-terminal active site). Abz-FRK(Dnp)P-OH (circles) was used as non-site specific substrate. Captopril (0.01 nM–100 nM, **A**) concentration-dependently inhibited serum ACE activity determined by all three substrates with a similar affinity. In contrast, serum fraction containing 50–100 kDa components (0.02–20 mg/mL protein concentration, 20 mg/mL represents 2.34-fold dilution, **B**) had higher affinity at the C-terminal active site (determined by Abz-LFK(Dnp)-OH). Symbols represent means ± SEM of 3 independent determinations, values are given in the percentage of control (without ACE inhibitor). Inhibitory activity of the serum fractions (proteins below 50 kDa, or in the range of 50–100 kDa, 2-fold dilution) and captopril (1 µM) were selectively tested by the Abz-FRK(Dnp)P-OH substrate (non-site specific flourescent substrate, **C**). Bars represent means ± SEM of the recombinant ACE activities in the percentage of vehicle (n = 3).

Serum ACE has two catalytically active domains. Application of specific fluorescent substrates for these active sites revealed that captopril have the same potency and efficacy on both active sites (Fig. 6A), while the endogenous serum inhibitor had higher potency on the C-terminal active site than that is for the N-terminal active site (Fig. 6B). Interestingly, the hydrolysis of the fluorescent substrate Abz-FRK(Dnp)P-OH (non-specific for the catalytic sites) was identical than that for the N-terminal site specific substrate in this latter case.

Recombinant ACE was also inhibited by the endogenous serum inhibitor (fraction 50–100 kDa) and captopril (Fig. 6C), when measured by the Abz-FRK(Dnp)P-OH, similarly to the angiotensin I (Fig. 5A) and FAPGG hydrolysis (Fig. 5B), suggesting that the ACE inhibition by the 50–100 kDa endogenous serum inhibitor is not a substrate specific feature. On the other hand, the serum fraction containing all of the components below 50 kDa (fraction <50 kDa) was again without effects on recombinant ACE activity on Abz-FRK(Dnp)P-OH hydrolysis, similarly to angiotensin I (Fig. 5A) and FAPGG hydrolysis (Fig. 5B).

**Figure 7:**
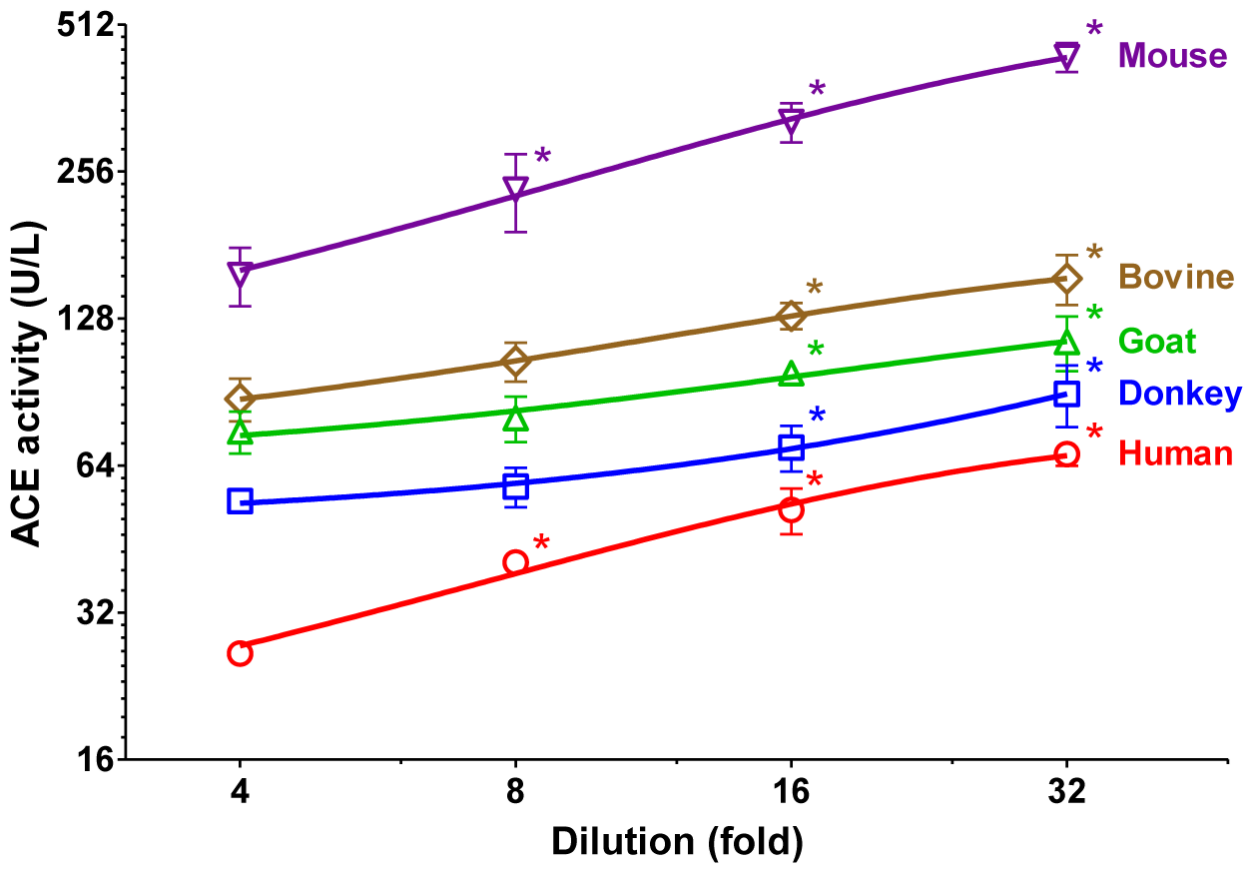
Endogenous serum ACE inhibition is evolutionary conserved. Effects of dilution on serum ACE activity were tested in different species. Specific ACE activities were plotted as a function of dilution levels. Symbols denote means ± SEM of at least 3 independent determinations. Significant differences from ACE activity values measured at 4-fold dilution are indicated by asterisks.

These data suggested that ACE is inhibited by a protein with an apparent molecular mass of 50–100 kDa in human serum. It was tested whether this inhibition is species dependent or an evolutionary conserved general feature. Normal serum samples from mouse, bovine, goat and donkey were tested and compared to human. Serum ACE activities were significantly different in these species ranging from 26.4±0.7 U/L in human to 157.2±12.5 U/L in mouse at 4-fold dilution. Nonetheless, specific ACE activities significantly increased upon dilution in each species (by 2.8-fold, 1.8-fold, 1.5-fold, 1.7-fold and 2.6-fold, respectively, Fig. 7).

**Figure 8:**
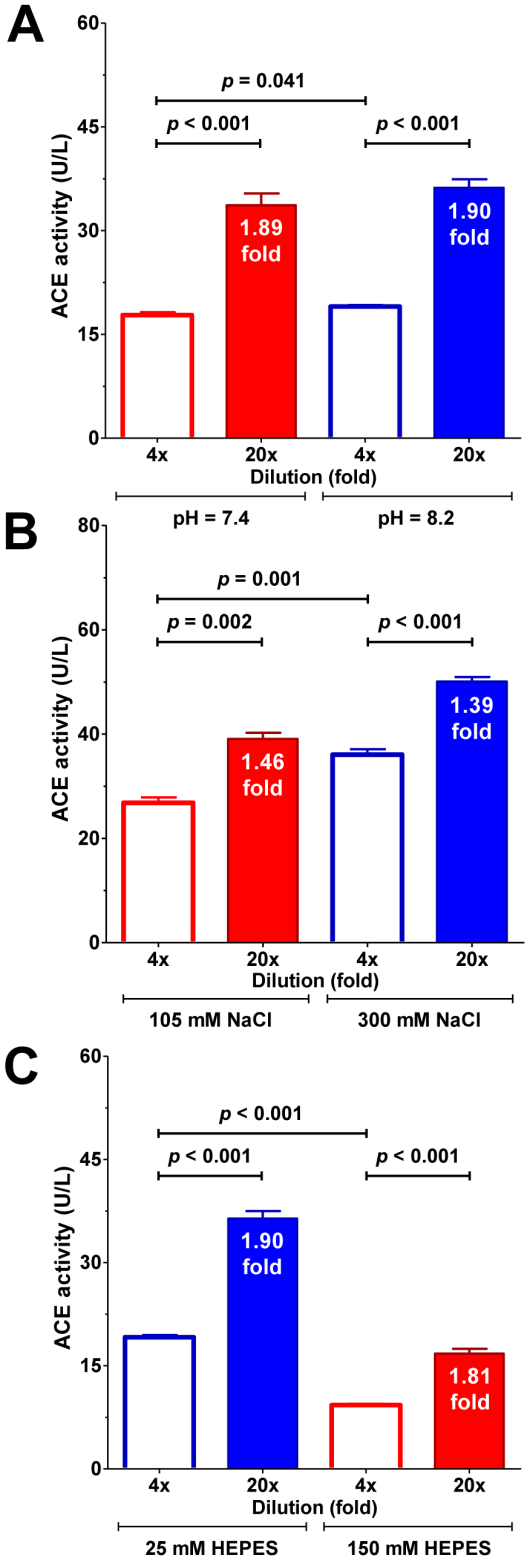
Differences in pH, ionic strength and buffer concentration do not effect the increase in ACE activities upon dilution. Effects of dilution (4 and 20-fold) were tested on ACE activity under different assay conditions. Increase in specific ACE activity was present at physiological pH (A), at physiological Cl^−^ (B) and at higher buffer concentrations (C). Blue bars represent optimal assay conditions used in the previous experiments. Bars denote means ± SEM of at least 3 determinations. Significant differences are indicated by the *p* values. The increase in ACE activities for each pairs are also shown within the bars.

The increase in specific ACE activity upon dilution was tested under different assay conditions. Increase in specific activity was present at physiological pH (Fig. 8A, increase in specific ACE activity by 1.89-fold at pH 7.4 versus 1.90-fold at pH 8.2) and Cl− concentrations (Cl− is essential for ACE enzymatic activity, Fig. 8B, increase in specific ACE activity by 1.46-fold at 105 mM NaCl versus 1.39-fold at 300 mM NaCl). Increase in specific ACE activity was not affected by the buffer concentration (Fig. 8C, increase in specific ACE activity by 1.9-fold at 25 mM HEPES versus 1.81-fold at 150 mM HEPES).

**Figure 9:**
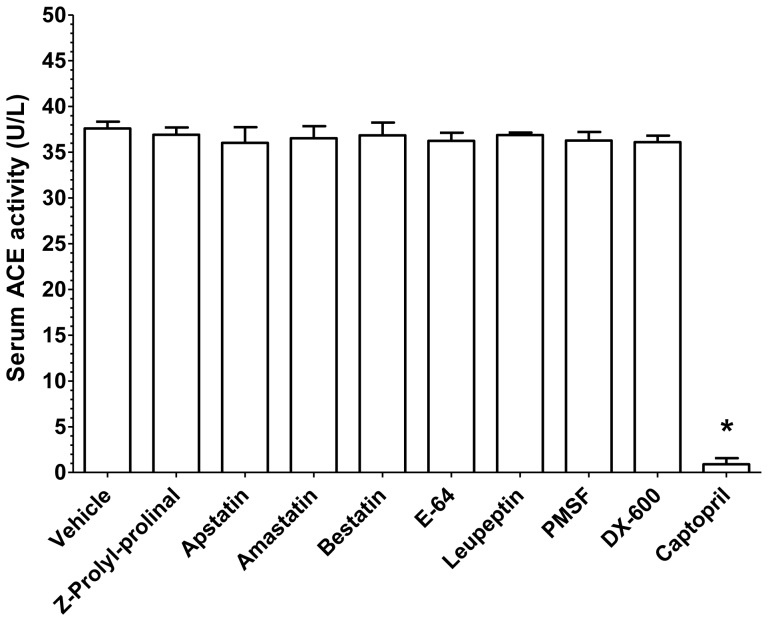
Effects of various protease inhibitors on FAPGG hydrolysis by human serum. Human serum (8-fold dilution) was incubated with Z-Prolyl-prolinal (prolyl-endopeptidase inhibitor, 1 µM), Apstatin (aminopeptidase P inhibitor, 10 µM), Amastatin (an inhibitor of various aminopeptidases, 10 µM), Bestatin (leucin aminopeptidase and aminopeptidase B inhibitor, 1 µM), E-64 (cysteine protease inhibitor, 1 µM), Leupeptin (serine and cysteine protease inhibitor, 10 µM), PMSF (serine and cysteine protease inhibitor, 100 µM), DX-600 (ACE2 inhibitor, 1 µM) and captopril (an ACE inhibitor, 1 µM) for 15 min. ACE activity measurement was initiated by the addition of FAPGG. Enzyme activity was measured for 120 min and apparent enzyme activities were expressed and plotted in absolute (U/L) units. Bars represent mean ± SEM of three independent determinations. Significant difference (*p<0.001*) from the control (vehicle) is indicated by the asterisk.

Finally, the specificity of FAPGG hydrolysis was also tested. A set of protease inhibitors (Z-Prolyl-prolinal, prolyl-endopeptidase inhibitor, 1 µM, Apstatin, aminopeptidase P inhibitor, 10 µM, Amastatin, an inhibitor of various aminopeptidases, 10 µM, Bestatin, leucin aminopeptidase and aminopeptidase B inhibitor, 1 µM, E-64, cysteine protease inhibitor, 1 µM, Leupeptin, serine and cysteine protease inhibitor, 10 µM, PMSF, serine and cysteine protease inhibitor, 100 µM, DX-600, ACE2 inhibitor, 1 µM) were without effects on FAPGG conversion by human serum (Fig. 9). On the other hand, captopril (an ACE inhibitor) successfully inhibited FAPGG conversion (enzyme activity decreased from 37.6±0.4 (vehicle) to 0.9±0.4 U/L, Fig. 9).

## Discussion

Here we propose that serum ACE is inhibited by an endogenous inhibitor under physiological conditions. This endogenous inhibitor is a protein with an apparent molecular weight of 50–100 kDa. Compared to competitive ACE-inhibitor drugs (like captopril), the endogenous factor is a non-competitive inhibitor, and it has higher potency on C-domain active site of ACE than N-domain active site.

Endogenous ACE inhibition is particularly interesting in light of the clinical effectiveness of ACE inhibitor drugs in cardiovascular diseases [4]–[10], as evidenced by several large-scale trials, and as accepted by the published guidelines [13]–[18]. Our data suggest that ACE activity is suppressed by the endogenous inhibitor, which may provide a protective mechanism for cardiovascular disease. An important aspect of our findings is that the reversible interaction between ACE and its inhibitor may provide a mechanism capable of stabilizing serum ACE activities at different levels of ACE concentration, similarly to conventional buffer systems which can stabilize the pH. According to this analogy, if the reversible inhibitor is in excess compared to ACE then the ACE is in its inhibitor bound form, irrespectively of slight variations in ACE concentration. Our data suggest that this provide an evolutionary conserved mechanism for quenching soluble ACE activity when it is shed from tissue sources, where it fulfills its biological function.

Indeed, serum ACE concentration is under genetic control in human. ACE insertion-deletion (I/D) polymorphism is responsible for 20–50% of the interpersonal variability in circulating ACE expression levels (II: 299.3±49 µg/L, ID: 392.6±67 µg/L, DD: 494.1±88 µg/L) [30]. According to these substantial differences in serum ACE activities it was proposed that the differences in ACE expression (related to ACE I/D polymorphism) may play a role in the occurrence of myocardial infarction, coronary artery disease, coronary artery calcification, heart failure and hypertension. However, most of the studies have failed to demonstrate any association between these diseases and ACE I/D polymorphism which is in accordance with our data [31].

One may argue that ACE I/D polymorphism determines not a 50% of blood ACE interpersonal variability as initially published by Rigat in 1990, but only about 20% [32] [33] or even 8% [34]. Therefore it is not surprising that association of ACE DD genotype with cardiovascular disease was not strong [35], in a sharp contrast to ACE phenotype [36]. Motivated by these results the effect of ACE I/D polymorphism on circulating ACE expression was tested in our Hungarian population [31]. A substantial effect of genotype was found on ACE expression (II: 101.0±6.7 µg/L, ID: 115.1±4.5 µg/L, DD: 157.7±6.3 µg/L), similarly to the original report by Rigat et al., making this population a prime target for further studies [31].

Irrespectively to the actual contribution of ACE genotype to ACE activity, a meta-analysis of more than 30,000 individuals led to the conclusion that ACE gene polymorphism does not affect blood pressure, and is not associated with an increased risk of myocardial infarction, ischemic heart disease or ischemic cerebrovascular disease [35]. Important to note that ACE inhibitors are primary used drugs to treat these diseases, suggesting a substantial contribution of ACE to these diseases. Moreover, there are mutations affecting more pronouncedly serum ACE concentration than the ACE ID polymorphism. Kramers et al. reported on cases involving a point mutation in the stalk region of the ACE gene, in which the amount of circulating ACE was 5-fold elevated [37]. This mutation affected at least eight families, but again, there were no ACE-related clinical abnormalities or hypertension. Nesterovitch et al. described another mutation that was accompanied by a 13-fold elevation of the serum ACE concentration, which again did not result in any disease [38]. All of these observations suggest that substantial differences in ACE concentrations are well tolerated. This may be explained by the presence of the reversible inhibitor stabilizing (buffering) ACE activity, *in vivo*, reported here.

An alternative explanation is that tissue level of ACE is 10–30 fold more than blood ACE, therefore tissue ACE participate more in cleavage of vasoactive peptides and even such increase in blood ACE do not contribute significantly in overall peptide hydrolysis by ACE [39]. However, we would like to point out that most of the data related to ACE “level” was determined by measuring ACE activity, instead of ACE concentration. Our data suggest that there may not be a direct relationship between ACE activity and expression. In particular, the endogenous inhibitor shown here may mask ACE activity in the serum, while it may not be present in the isolated tissues, resulting in high apparent ACE “levels” in the tissues and low apparent ACE “levels” in the serum. ACE “levels” may represent simply those ACE pools, where ACE is not inhibited effectively, under the conditions used to isolate ACE.

As a matter of fact one of the implications of this study is that ACE activity is controlled by endogenous inhibitors. Circulating ACE may be suppressed, irrespectively to its concentration. In this respect, circulating ACE may be a pool of inactivated ACE. One may speculate that the evolutionary function of the endogenous ACE inhibition is to neutralize ACE when it is shed from the tissue, to make sure that it does not interfere with its physiological function in the tissues. To validate these implications new human trials are necessary. In particular, we have proven that the endogenous inhibition in the sera shown here can suppress differences in ACE concentrations, such as reported in relation to the ACE genotype [31].

Regarding the existence of endogenous ACE inhibiting factors there are some important earlier reports in the literature. Existence of endogenous ACE (called kininase II at this time) inhibitors were proposed as early as 1979. Klauser et al. reported that plasma preparations and human serum albumin inhibit the ACE [20], which was also confirmed by us [40] while Ryan et al. reported that small (<10 kDa) molecular weight components of human sera and urine are also able to inhibit purified ACE [21]. These original reports were followed by some reports suggesting that substrate analogues (such as angiotensin I [22] and substance P [23]) can also inhibit ACE. The existence of endogenous ACE inhibitors were confirmed later. Lieberman et al. reported an ACE inhibitor with an apparent molecular mass of >50 kDa [24]. Ikemoto et al. reported the presence of an other sulfhydryl (SH) specific endogenous inhibitor, with an apparent molecular mass of >10 kDa [25]. C-type natriuretic peptide was also proposed to have indirect inhibitory effects on ACE activity, although the exact mechanisms were not revealed [41]. Identification of the synthetic ACE inhibitors as a new class of drugs acting on blood pressure [42] gave rise to the technique which made it possible to affinity purify ACE (captopril or lisinopril affinity chromatography), together with its interaction partners. It was revealed that ACE associates with both low and high molecular weight ACE inhibitors in the rat lung [26] or with a 14 kDa inhibiting protein in the human sera [27]. The clinical success of the synthesized ACE inhibitors also facilitated the identification of dietary factors with ACE inhibiting properties, such as peptides of the tryptic lisate of human plasma [43], [44], human serum albumin [45], bovine alphaS2-casein [46]. Some of the beneficial effects of honey were also related to ACE inhibitory activities [47].

Our data do not support the existence of low molecular weight ACE inhibitors in the sera. Serum fractions containing components under 50 kDa did not show any inhibitory effects on serum or recombinant ACE activity. The possible explanation can be the connection of these inhibitors to diet. Blood sample collections were executed after overnight fasting period of the healthy volunteers, which probably enabled the elimination of low molecular weight inhibitors from the circulation. It is also possible that some of these small molecular weight inhibitors are the result of proteolytic degradation of the high molecular weight inhibitor(s) and that this proteolysis did not occur in our samples. Finally, it is also possible that we have used to high dilution factors to detect these small molecular weight regulators of human circulating ACE.

These findings suggest that the determination of ACE activities can be complicated by the endogenous inhibitor. Indeed, an earlier work suggested an 8-fold dilution to get rid of the inhibitory effect in ACE activity measurements [24]. Dilution may be a good technique to eliminate the effects of endogenous inhibitors to obtain reliable ACE activity values, but it may cause some confusion in the interpretation of the data. In particular, physiological ACE activity may be significantly overestimated. We have demonstrated here that physiological ACE activities are at least 2.8-fold overestimated when *in vitro* determination is done at high dilutions. Note, that this value (2.8-fold overestimation) represents the minimum factor by which ACE is physiologically inhibited. Indeed, it has been proven in accompanying papers that physiological ACE activity is much lower than one third of the measured ACE activity [31], [40].

Our findings suggest that this endogenous inhibitor has a relatively low dissociation constant, because its effect can be easily eliminated using low dilutions of serum samples. Thus the isolation of this inhibitor is a great challenge, considering it will be lost during washing steps of purification.

The main novel information in the current study is: (1) FAPGG and angiotensin I conversions are closely related, and therefore the endogenous inhibition is probably not an artifact resulting from the use of the artificial substrate; (2) The increase in ACE activity by dilution is not present when ACE is recombinantly expressed; (3) Endogenous inhibition of ACE is an evolutionary conserved mechanism; (4) The endogenous ACE inhibition is non-competitive; (5) The endogenous ACE inhibitor has a higher affinity on the C-terminal active site of the ACE.

**Figure 10:**
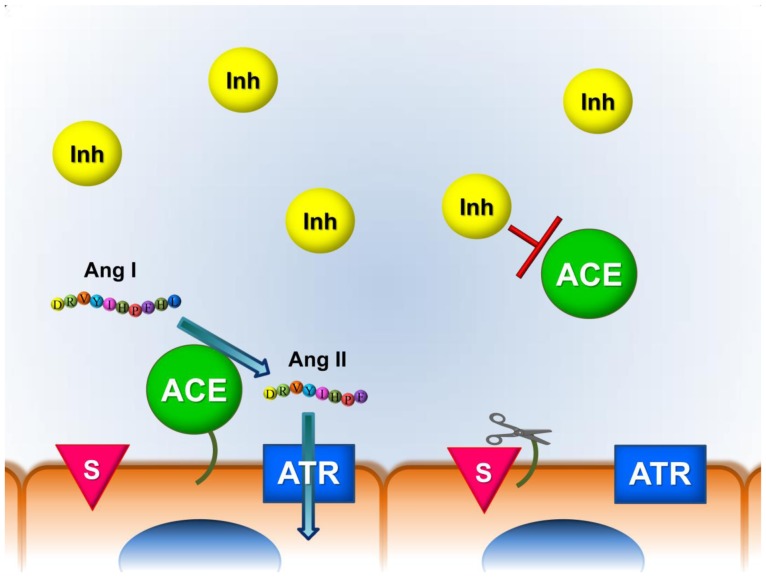
Hypothetical function of the endogenous angiotensin converting enzyme (ACE) inhibitor. The clinical success of ACE inhibitory drugs prove that ACE is a physiologically important angiotensin I converting enzyme. Here we propose the existence of an endogenous ACE inhibitor in human sera (Inh) which provide an evolutionary conserved mechanism for the suppression of circulating ACE activity. We hypothesize that ACE is catalytically active in the tissues, where it converts angiotensin I to angiotensin II. The formed angiotensin II then binds to its resident receptors (ATR) and activates intracellular signal transduction leading to physiological responses. Soluble ACE is the result of the shedding of tissue-bound ACE mediated by the ACE secretase (S). Our data suggest that this soluble ACE activity is inhibited by an endogenous inhibitor (Inh), restricting ACE mediated angiotensin I conversion to the tissues, irrespectively to the concentration of the circulating ACE. ACE function appears to be quenched by the endogenous inhibitor when it is secreted into the systemic circulation. This mechanism may contribute to the confinement of angiontensin II mediated physiological responses.

Based on these findings we propose that the activity of circulating ACE, shedded into the serum, is inhibited by an endogenous inhibitor providing an evolutionary conserved mechanism for suppressing circulating ACE activity (Fig. 10).
